# Tenderness on the bicipital groove: is it an indication for biceps procedure during rotator cuff repair?—A prospective randomized controlled study

**DOI:** 10.1016/j.jseint.2025.09.009

**Published:** 2025-10-15

**Authors:** Seong-Jun Kim, Jae-Hyung Lee, Kyung-Soo Oh, Jin-Young Park

**Affiliations:** a Joint Center Department of Orthopedic Surgery, Seoul Bumin Hospital, Seoul, Republic of Korea; b Cheongdam Reon Orthopaedic Clinic, Seoul, Republic of Korea; c Glocal Center for Shoulder & Elbow, Department of Orthopedic Surgery, Konkuk University Hospital, Konkuk University School of Medicine, Seoul, Republic of Korea; d Center for Shoulder, Elbow and Sports Medicine, Neon Orthopaedic Clinic, Seoul, Republic of Korea

**Keywords:** Tenodesis, Tenotomy, Biceps, Rotator cuff, Bicipital groove, Rotator cuff repair

## Abstract

**Background:**

Treating long head of biceps lesions associated with rotator cuff tears is a major issue, and pain alone without any other structural lesion may be an indication for treating biceps. The purpose of this study was to evaluate the advantages of tenodesis in patient with symptom of pain on the bicipital groove without biceps lesions during rotator cuff repair.

**Method:**

This study included 90 patients [38 males, 52 females; average age, 59.19 (range, 42-74) years] who underwent surgery for rotator cuff tears (small- to medium-sized) between February 2020 and August 2021. All patients had pain on the bicipital groove and no biceps tendinopathy (complete tear, partial tear, severe tenosynovitis, superior labrum anterior and posterior lesion (II, III, IV), and subluxation). Patients were randomly assigned to either the tenodesis group or the no-specific treatment group based on the parity of their enrollment number. Patients with odd-numbered enrollment were allocated to the tenodesis group (T) (n = 45), while those with even-numbered enrollment were assigned to the no-treatment group (N) (n = 45). The mean follow-up duration was 28.53 (range, 24-44) months. Preoperative and postoperative (24 months) visual analog scale (VAS) scores and American Shoulder and Elbow Surgeons (ASES) scores were calculated. Also, pain on bicipital groove, effusion, injection on the bicipital groove, and presence of Popeye deformity was checked.

**Results:**

Both groups demonstrated significant improvements in VAS and ASES scores at 24 months. However, the between-group differences did not exceed the minimal clinically important difference (VAS difference = 0.58, ASES difference = 1.13; both *P* > .05). Persistent bicipital groove pain was significantly more frequent in the N group (24.4%) compared to the T group (2.2%, *P* < .01). The N group also had a higher incidence of postoperative corticosteroid injection (17.8% vs. 2.2%, *P* < .03). No Popeye deformities were observed in either group.

**Conclusion:**

Although pain, VAS score, and ASES score were improved in both groups, treating bicipital groove pain with tenodesis during rotator cuff repairs in patients with pain on the bicipital groove is effective on reducing pain and injection after operation.

The long head of the biceps tendon (LHBT) has a unique anatomy,^9^ but its functional role in glenohumeral joint stability is not well understood.^5^ Although the functional significance of the LHBT remains a topic of debate, the LHBT is recognized as a cause of anterior shoulder pain.^7^ Treatment of long head of biceps lesions associated with rotator cuff tears is an important issue, and pain alone, without other structural lesions, may be an indication for biceps treatment.^6^

Biceps brachii long head tendinopathy is a very common pathology in patients with anterior and deep shoulder pain.^10^ It has also been associated with rotator cuff tears and superior labrum anterior and posterior (SLAP) lesions due to changes in loading.^4^

LHBT lesions have been reported to affect approximately 70% of patients with rotator cuff tears.^5^ Morphological changes were observed in 19.8% of untreated asymptomatic LHBT even after rotator cuff repair.^8^ Even after successful rotator cuff repair, the biceps tendon in the biceps groove thickens over time. In addition, an increase in blood vessels around the biceps tendon in the groove was observed; therefore, LHBT treatment may be necessary during rotator cuff surgery.^5^

The purpose of this study was to evaluate the advantages of tenodesis in patients with symptoms of pain (tenderness) on the bicipital groove without any anatomical biceps lesions during rotator cuff repair. The authors hypothesized that even if patient has no structural lesions, there would be persistent pain when the patient with bicipital groove pain did not receive any biceps procedure.

## Material and methods

### Patient allocation/inclusion and exclusion criteria

**Figure 1:**
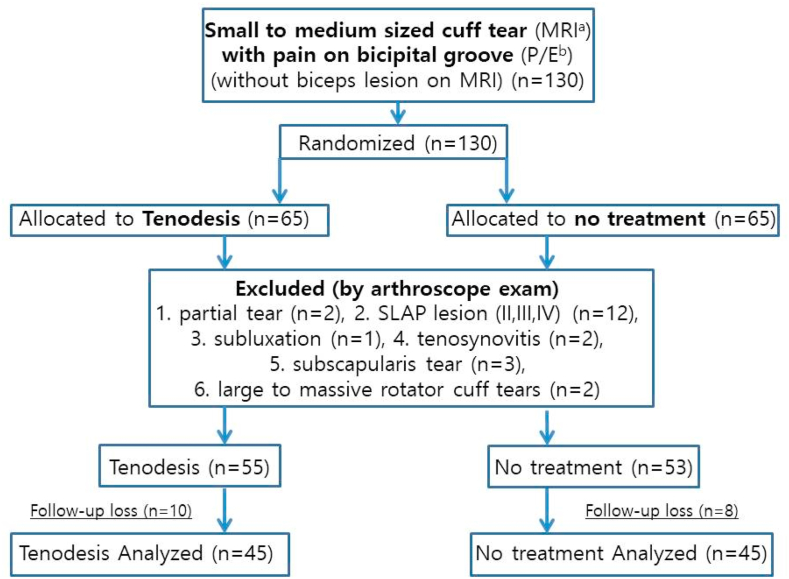
Flow diagram of the trial. *MRI*, magnetic resonance imaging; *P/E*, physical examination; *SLAP*, superior labrum anterior and posterior.

This was a prospective randomized controlled study. Institutional review board approved this study (KUH1060182), and all patients were provided with written informed consent prior to participation in this prospective clinical trial. All of the patients enrolled in this study had tenderness on the bicipital groove on physical examination and were diagnosed with small to medium rotator cuff tears by preoperative magnetic resonance imaging, and concurrently diagnosed with no long head of biceps lesion by arthroscopic inspection between February 2020 and August 2021. Long head of biceps lesions were defined as complete tear, partial tear, severe tenosynovitis, SLAP lesion (II, III, IV), and subluxation. Patients were randomly assigned to either the tenodesis group or the no-specific treatment group based on the parity of their enrollment number. Patients with odd-numbered enrollment were allocated to the tenodesis (bridge tenodesis^16^) group (T), while those with even-numbered enrollment were assigned to the no-treatment group (N). Initially, 130 patient were prospectively enrolled in this study, and 40 patients were excluded because of loss of follow-up (n = 18), partial tear of the biceps (n = 2), SLAP lesion (II, III, IV) (n = 12), long head tendinopathy subluxation (n = 1), tenosynovitis (n = 2), subscapularis tendon tear (n = 3), and large to massive rotator cuff tears (n = 2) (Fig. 1). Finally, 90 patients [38 males, 52 females; average age, 59.19 (range, 42-74) years]—tenodesis group (T) (n = 45) and the no-specific treatment group (N) (n = 45)—were analyzed (Table I).

**Table I:** Patient demographics and clinical data.

Group	Tenodesis (T) (n = 45)	No treatment (N) (n = 45)	*P* value
Age, yr	60.67 ± 6.15 (48-74)	57.71 ± 7.33 (42-72)	.090
Sex			.134
Male	15	23	
Female	30	22	
Follow-up, mo	28.20 ± 4.63 (24-40)	28.87 ± 5.22 (24-44)	.524
Size			.318
Small	32	37	
Medium	13	8	
Preoperative VAS	5.67 ± 2.33 (2-10)	4.36 ± 1.75 (1-9)	.365
Preoprative ASES	55.89 ± 22.16 (16-90)	60.47 ± 16.46 (10-88)	.364
Anchors used	3.27 ± 0.58	3.09 ± 0.29	.117

*VAS*, visual analog scale; *ASES*, American Shoulder and Elbow Surgeons.

## Surgical procedure

All of the operation was conducted at a single center, with indications of pain on the bicipital groove on physical examination, small to medium rotator cuff tears by preoperative magnetic resonance imaging, and concurrently diagnosed with no long head of biceps lesions (complete tear, partial tear, severe tenosynovitis, SLAP lesion (II, III, IV), and subluxation), as mentioned above. Although the area beneath the bicipital groove could not be examined, the absence of pathology was confirmed by pulling the tendon within the glenohumeral joint.

Under general anesthesia, patients were placed in a “beach-chair” position (lift-assisted beach-chair positioner; Arthrex, Naples, FL, USA), with the patient’s head securely fixed to a rigid helmet. Sterilization of the patient’s skin with 2% chlorhexidine was done and draping was performed using sterilized shoulder pack (Yuhan-Kimberley, Seoul, Republic of Korea). An arthroscopic device and a 30° angled camera (Synergy^HD3^; Arthrex, Naples, FL, USA) was used for the operation.

### Routine intra-articular procedure/subacromial decompression

Bony land mark was neatly drawn to identify the anatomic landmarks. At first, posterior portal into the glenohumeral joint was made for routine arthroscopic inspection. Using an 18-gauge needle to find a proper spot at the rotator interval, an anterior portal was made, and probe was introduced inside the joint to assist inspection of intra-articular lesions. During the intra-articular inspection, if there were presence of biceps lesions (complete tear, partial tear, severe tenosynovitis, SLAP lesion (II, III, IV), and subluxation), authors had dropped the cases from the study.

After completing the intra-articular procedure, scope was introduced to subacromial space for bursal space inspection and procedures. Meticulous acromioplasty was done and excessive bursal tissue was removed for clear visualization.

## First step of tenodesis: soft tenodesis (fixation on rotator interval tissue)

**Figure 2:**
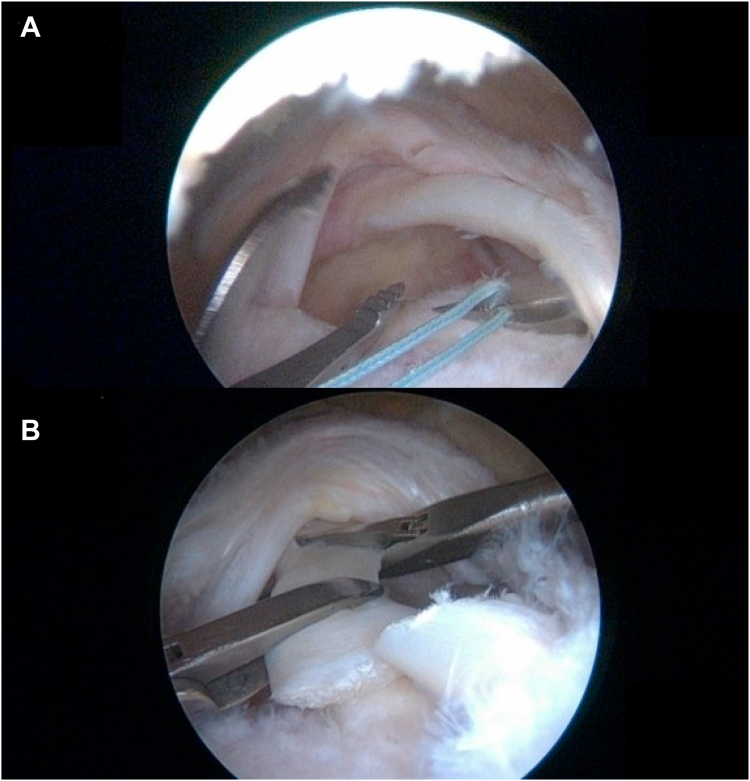
First step of tenodesis: soft tenodesis. (**A**) Soft tenodesis on the bursal side of the rotator interval. (**B**) Post soft tenodesis and biceps tenotomy.

After placing an anterior cannula (6.5 mm) (ConMed, Largo, FL, USA) and a posterior cannula (8 mm) (ConMed) to make passages for the instruments, a soft-tissue tenodesis of the long head of the biceps was performed. A straight BirdBeak suture passer (Arthrex, Naples, FL, USA) is passed through the rotator interval and the biceps tendon just before the starting point of the proximal bicipital groove using a fiber wire (Arthrex, Naples, FL, USA) (Fig. 2, *A*). After the use of the suture passer, the next stop requires passing of a loose fiber wire between the LHBT and the rotator interval tissue. With the use of nonsliding knots, rigid fixation was achieved, with particular emphasis on the bursal side of the rotator interval tissue. After tying up, loose fiber wires were cut with a suture cutter. Using arthroscopic scissors, the biceps tenotomy was performed just proximal to the soft tenodesis site (Fig. 2, *B*).

### Medial row fixation

Next, the rotator cuff repair was performed. A medial row fixation was carried out by inserting an anchor (Triple Play; Arthrex, Naples FL, USA) at the medial margin of the greater tuberosity footprint or the lateral margin of the humeral cartilage junction. A suture hook (Linvatec, Largo, FL, USA) was used to pass the fiber wires from the medial anchor through the rotator cuff tendon, and an anatomical restoration of the rotator cuff was made. We conventionally performed a suture bridge technique to maximize contact pressure on the greater tuberosity footprint.

## Second step of tenodesis: bony tenodesis (fixation at the bicipital groove)

**Figure 3:**
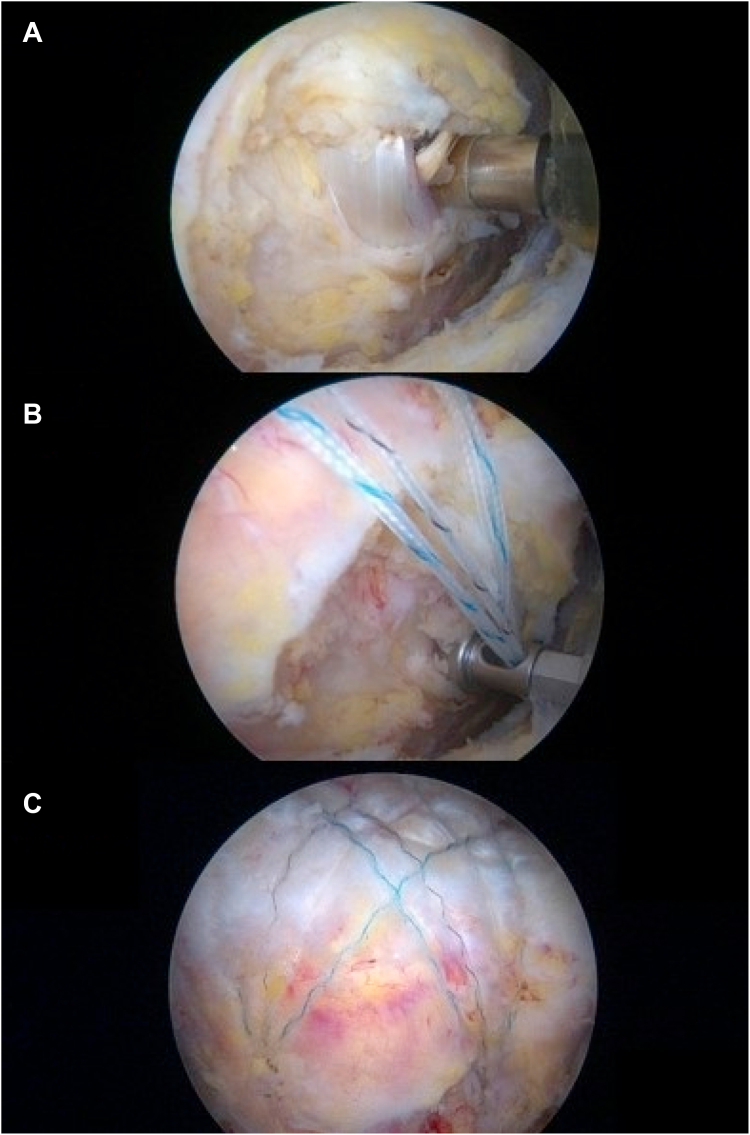
Second step of tenodesis: bony tenodesis. (**A**) Identification of biceps at the bicipital groove. (**B**) Lateral knotless anchor was placed at bicipital groove. (**C**) Tenodesis of the long head of biceps tendon.

After medial-row tying, identification of biceps at the bicipital groove was done using a radio frequency device (Arthrocare, Austin, TX, USA) (Fig. 3*A*). Then, the second part of bridge tenodesis, which is bony tenodesis, was performed. The lateral knotless anchor (4.75-mm Swivelock SP; Arthrex, Naples, FL, USA) was placed at bicipital groove (Fig. 3*B*), purchasing the long head of biceps tendon (Fig. 3*C*). The reasons for using self-punching lateral anchor were to make a better penetration and make less damage to long head of biceps tendon. The other lateral anchors were inserted posterior to the first lateral anchor (on the bicipital groove), depending on the tear configuration.

The aim of this technique was to make a secure fixation of the biceps during rotator cuff repair by making 2 tenodesis sites (rotator interval and bicipital groove). The suture materials in between the both tenodesis site makes a bridge, therefore, the authors named it bridge tenodesis.

### Rehabilitation

After the operation, an abduction sling brace was applied for 6 weeks, and simultaneously passive motion (pulley device exercise) of forward elevation was allowed. After 6 weeks, passive-assisted exercise (external rotation, forward elevation, and abduction–external rotation) was carried out. In 10 weeks after operation strengthening exercises (thera-bands) were done, and at 3 months, unrestricted daily activity was allowed. Return to sports was allowed at 6 months (except contact and collision sports), and at 9 months, contact and collision sports were allowed, depending on the patient’s recovery.

### Evaluation

Authors have evaluated several parameters to check the efficacy between the 2 groups: the bridge tenodesis group (T) and the no-specific treatment group (N). First, preoperative and postoperative (at 24 months of follow-up) visual analog scale (VAS) score and American Shoulder and Elbow Surgeons (ASES) score were checked through questions and physical examination at the clinic. Then pain on the bicipital groove was checked by tenderness of the patients, through direct physical examination. The effusion was checked via ultrasound image and presence of Popeye deformity was check under direct visualization. Lastly, injection history on the bicipital groove was investigated.

### Statistical analysis

Statistical analysis was performed with SPSS 12.0 software (IBM Corp., Armonk, NY, USA). The statistical significance was set at *P* < .05. *T*-test test was used to check the validity of follow-up period, age, VAS, and ASES. Chi-square test was used to calculate the validity of follow-up period, age, VAS, and ASES between 2 groups. Also, Chi-square test was used to compare the parameters of pain on bicipital groove, effusion, injection history, and presence of Popeye deformity.

## Results

**Figure 4:**
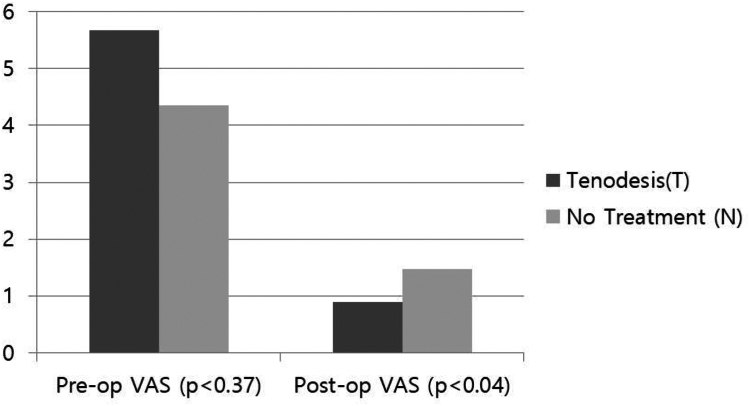
Preoperative–postoperative VAS score. *VAS*, visual analog scale.

**Figure 5:**
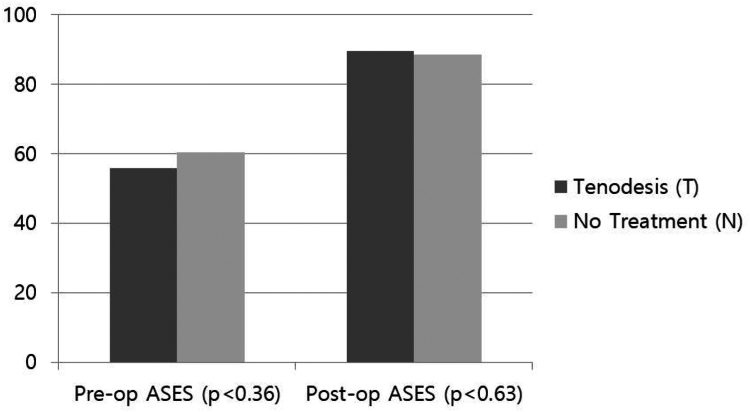
Preoperative–postoperative ASES score. *ASES*, American Shoulder and Elbow Surgeons.

At 24 months follow-up, final VAS score compared to preoperative VAS score decreased in both groups (T group: 5.67- 0.89, N group: 4.36- 1.47) (preoperative VAS, *P* < .37/final VAS, *P* < .04) (Fig. 4). ASES scores improved in both groups (T group: 55.89- 89.53, N group: 60.47-88.40) (Fig. 5). However, the statistical difference between the groups was not significant (*P* > .05) for VAS and the ASES scores at latest follow-up. (Table II).

**Table II:** Results.

Group	Tenodesis (T) (n = 45)	No treatment (N) (n = 45)	*P* value
VAS			
Preoperative VAS	5.67 ± 2.33 (2-10)	4.36 ± 1.75 (1-9)	.365
Postoperative VAS	0.89 ± 0.88 (0-3)	1.47 ± 1.59 (0-5)	.037
ASES			
Preoperative ASES	55.89 ± 22.16 (16-90)	60.47 ± 16.46 (10-88)	.364
Postoperative ASES	89.53 ± 10.62 (60-100)	88.40 ± 11.53 (45-100)	.063
Pain (tenderness)	1	11	.003
Effusion (by US)	5	6	.090
Injection	1	8	.029
Popeye deformity	0	0	N/A

*VAS*, visual analog scale; *ASES*, American Shoulder and Elbow Surgeons; *US*, ultrasound.

**Figure 6:**
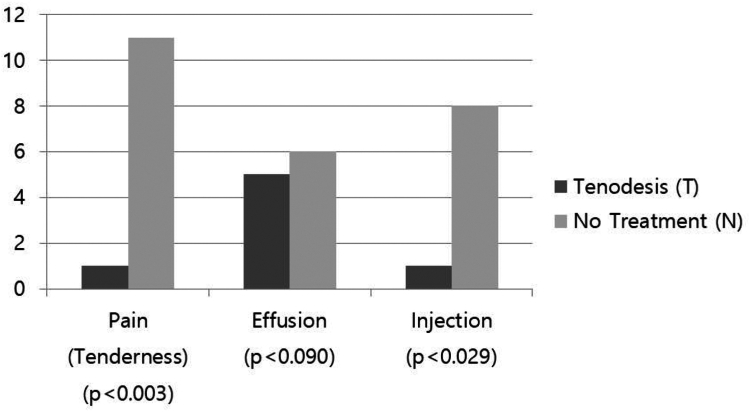
Postoperative clinical features.

In T group, 1 patient had pain on bicipital groove, 5 patients had effusion signs, injection was performed in 1 patient, and no Popeye deformity was occurred. In N group, 11 patients had pain on bicipital groove, 6 patients had effusion signs, injection was performed in 8 patients, and there was no Popeye deformity. At the 24-month follow-up, 24.4% (11 out of 45) of patients in the no-specific treatment (N) group continued to experience bicipital groove pain, whereas only 2.2% (1 out of 45) of patients in the tenodesis (T) group reported persistent symptoms. The need for postoperative corticosteroid injections was markedly higher in the N group (17.8%, 8 out of 45) compared to the T group (2.2%, 1 out of 45) (*P* < .03), reinforcing the analgesic benefits of tenodesis. Although the effusion sign was shown marginal significant between 2 groups (*P* < .09), pain on bicipital groove (*P* < .01) and injection history (*P* < .03) were significantly higher prevalence in N group (Fig. 6).

## Discussion

At a minimum 24-month follow-up, both groups showed improvement in VAS and ASES scores; however, the between-group differences did not exceed the minimal clinically important difference, approximately 1.5-2.0 points for VAS and 6-12 points for ASES, indicating limited clinical relevance.^11^^,^^12^ Comparing prevalence of complications of 2 groups, the prevalence of pain on bicipital groove (*P* < .01) and injection history (*P* < .03) were significantly higher prevalence in N group. This study demonstrates that performing biceps tenodesis in patients presenting with bicipital groove pain may result in significant postoperative pain reduction and decreased need for additional interventions.

The results of this study are shown that bicipital groove pain can be caused surgical intervention, and tenodesis and tenotomy in both group is shown decreasing pain and less prevalence of complications as an effective alternative method at minimum 24 months follow-up. This finding is corresponded with other studies. Zhu et al^17^ performed meta-analysis of level I evidence comparing tenotomy vs. tenodesis of LHBT using 5 studies (227 tenotomy and 227 tenodesis patients) and reported that both surgeries resulted in comparable postoperative clinical and functional outcomes. A Popeye deformity complication is less likely to occur with tenodesis than tenotomy. Belk et al^1^ performed a systematic review and meta-analysis of level I randomized controlled trials using 5 studies (236 tenodesis and 232 tenotomy patients), with 23 months of mean follow-up. They reported that no differences in VAS or ASES were found between both groups. They concluded that patients undergoing treatment for LHBT or SLAP pathology with either biceps tenodesis or tenotomy can be expected to experience similar improvements in patient-reported and functional outcomes.

Although the role of the LHBT in shoulder biomechanics and pathology remains controversial,^13^^,^^15^ it is widely recognized as a potential pain generator in patients with rotator cuff tears.^2^^,^^14^ Previous studies have demonstrated that persistent bicipital groove pain due to ongoing inflammation around the tendon may occur even following successful rotator cuff repair. Chen et al,^3^ in arthroscopic studies evaluating 122 shoulders, reported that 76% of patients with rotator cuff disease LHBT lesions in the form of tendinitis and subluxation were seen. These include dislocation, partial rupture, and complete rupture. These changes are both visually and microscopically evident in LHBT. Elsewhere, it was positively associated with severity of the rotator cuff tear. In this study, abnormality of LHBT during rotator cuff repair procedure were consistently found and performed concomitant surgeries such as tenotomy or tenodesis.

This study has several limitations. First, although it was designed as a prospective randomized controlled trial, the randomization was performed using a simple odd-even assignment based on the order of enrollment. While this ensured equal group sizes, it lacked allocation concealment and may have introduced selection bias. Blinding was also not implemented.

Second, a formal power analysis was not conducted prior to the study because of the lack of comparable previous research on this specific patient population. As a result, the study may have been underpowered to detect clinically meaningful differences in outcome scores.

Third, data were collected at only 2 time points: before surgery and at 24 months postoperatively. The absence of intermediate follow-up limits the ability to assess the clinical course and the timing of any changes in symptoms or function.

Fourth, although patients with visible biceps pathology were excluded, subtle degenerative changes that could not be identified arthroscopically may have affected the results. Future studies using advanced imaging modalities could better evaluate the contribution of occult biceps pathology.

## Conclusion

This study provides evidence that biceps tenodesis significantly reduces bicipital groove pain in patients undergoing rotator cuff repair, even in the absence of identifiable structural LHBT lesions. Given that nearly one-quarter (24.4%) of patients who did not undergo tenodesis continued to experience persistent pain at 24 months postoperatively, surgeons should consider biceps tenodesis in individuals presenting with bicipital groove tenderness, regardless of arthroscopic findings. However, further long-term studies are required to validate these findings and optimize clinical decision-making in the management of biceps-related shoulder pathology.

## Disclaimers:

Funding: No funding was disclosed by the authors.

Conflicts of interest: The authors, their immediate families, and any research foundation with which they are affiliated have not received any financial payments or other benefits from any commercial entity related to the subject of this article.
